# The Utility of Social Media on Urology Residency Doximity Rankings

**DOI:** 10.7759/cureus.29666

**Published:** 2022-09-27

**Authors:** Shannon J Leung, Benjamin J Chiang, John T Roseman, Adam Klausner

**Affiliations:** 1 Urology, Virginia Commonwealth University School of Medicine, Richmond, USA; 2 Urology, University of Texas Health Science Center at Houston, Houston, USA; 3 Surgery, Riverside University Health System Medical Center, Riverside, USA; 4 Urology, Virginia Commonwealth University Health System, Richmond, USA

**Keywords:** covid, doximity, residency ranking, residency recruitment, urology residency, social media

## Abstract

Background

Social media has been more widely used by urology residency programs since the COVID-19 pandemic. There are no studies on the relationship between Doximity residency ranking and social media usage in urology.

Objectives

In this study, we aim to investigate the relationship between social media usage and the academic prestige of urology residency programs.

Methods

Residency reputation data was acquired from the Doximity Residency Navigator website. Twitter and Instagram activity in 2019 and 2021 was analyzed by collecting data on the total number of posts and followers. Data on residency virtual recruitment was obtained from Twitter and UroResidency website.

Results

By the end of 2021, 122/139 (87.8%) urology residency programs had a Twitter account and 61/139 (43.9%) had an Instagram account. A significant linear regression was found between Doximity ranking and the number of Twitter followers (p<0.001), Twitter posts (p=0.005), and Instagram followers (p=0.026). Virtual recruitment events were held by 107/139 (77%) programs in 2021. There was a significant linear regression between Doximity rankings and the number of virtual events (p<0.006).

Conclusions

Social media use by urology residency programs has increased since the COVID-19 pandemic. A program’s higher Doximity ranking was correlated with the presence of Twitter and Instagram accounts as well as the number of Twitter followers, Twitter posts, and Instagram followers. There was a significant relationship between Doximity rankings and the number of hosted virtual events. Programs should consider increasing social media visibility to potentially improve their Doximity rankings.

## Introduction

Social media use has become more prevalent and important in the field of academic urology. In 2014, the American Urological Association (AUA) reported that 71% of its members used some form of social media [1]. Twitter has been increasingly used by urological journals to report important guideline updates and highlight significant articles [2]. In addition, 77% of urology residency program directors reported using Twitter in 2021 [3]. Instagram was reported in 2017 to be the fastest-growing social media platform among urologists [4]. The use of social media in urology residency programs has significantly increased since 2020, likely as a result of the COVID-19 pandemic [5]. In 2020 alone, 23 urology programs created Twitter accounts, the largest single-year increase since 2009 [5]. Both applicants and residency program directors in the 2020-2021 AUA Match cycle found social media useful [3,6]. Social media has been an important tool for programs to advertise virtual residency recruitment events, which the majority of urology applicants found to be helpful in the 2020-2021 AUA Match cycle [7].

There have been a few studies analyzing the use of social media with the AUA Match outcomes [3,6], but there are currently no studies in urology specifically correlating social media usage with measures of residency program reputation, such as Doximity ranking, which has often been considered a surrogate for academic prestige [8]. Doximity is an online professional networking platform for healthcare professionals that provides numerous services including the Residency Navigator, which provides a specialty-specific database for prospective residency applicants. A survey conducted in 2018 reported that 62% of residency applicants used Doximity during the application cycle, and of those applicants, 78% found Doximity rankings to be valuable [9]. The relationship between Doximity residency ranking and social media usage has been previously demonstrated in orthopedic surgery and otolaryngology, but not for urology [10,11].

With travel restrictions and away rotation limitations due to COVID-19 making it more challenging for prospective residents to explore residency programs [3], programs’ use of social media has become more prevalent, especially for recruiting purposes [12]. As a result of this rapidly expanding use of social media by the urological academic community, we hypothesize that the presence and activity of urology residency Twitter and Instagram accounts have significantly increased between 2019 and 2021. We also hypothesize that this increased presence and activity on social media may correlate with a urology residency program’s ranking on Doximity. Lastly, we hypothesize that there may be a relationship between the number of virtual events and a program’s Doximity ranking.

## Materials and methods

A comprehensive list of urology residency programs in the United States was acquired and ranked 1 to 145 based on the Doximity Residency Navigator, with 1 being the highest ranked [13]. Programs were also categorized based on geographic sections of the AUA [14]. Programs that did not recruit residents for either the 2019-2020 or the 2021-2022 cycles and military-affiliated programs were excluded from the analysis. Data were collected from January 7, 2022, to January 12, 2022.

Two independent reviewers used the Google search engine with the search term “name of institution + urology residency” + “social media account” (e.g., (name) urology residency Twitter) to determine the presence of a social media account. Reviewers also used the Association of American Medical Colleges’ Electronic Residency Application Service and official urology departmental websites to identify social media accounts [14,15]. All identified social media accounts were public. Identities of accounts were verified by identifying at least two affiliated physicians among the accounts’ followers. For programs that owned Instagram and Twitter, the reviewers further analyzed the number of original posts made by each account in the years 2019 and 2021. Retweets, comments, or replies were excluded from the total count because they are not primarily authored. These modalities are typically used to continue a conversation or thought and therefore were not considered as more than one instance of digital engagement in this study. In addition, the number of followers and the number of following were collected for each program for both Twitter and Instagram.

If a program had multiple accounts identified within the same social media platform, the account with more followers was used in the statistical analysis. Finally, for the 2021-2022 application cycle, the presence and number of virtual residency recruitment events (e.g., open houses, meet and greets, and happy hours) were collected using Twitter and UroResidency, a urology residency application advisement website, and analyzed against Doximity ranking.

Since the variable of interest in social media usage was the COVID-19 pandemic, the years 2019 and 2021 were chosen as the proxy for “before the pandemic” and “after the pandemic,” respectively. The year 2021, rather than 2020, was chosen for comparison because COVID-19 was not declared a pandemic until March 11, 2020, and the first stay-at-home order in the United States was not ordered until March 19, 2020 [16,17].

The Shapiro-Wilk test was run to assess for normality of all study distributions, and the appropriate statistical analyses were then performed. Significance was set at p<0.05. All statistical analyses were performed on Statistical Package for the Social Sciences (SPSS) version 28.0 (IBM Corporation, Armonk, NY, USA). This study was deemed Exempt Category 4 by the Institutional Review Board (IRB).

## Results

Social media among urology residency programs

A total of 145 urology programs were available on the Doximity Urology Residency Navigator, of which 139 programs were included. Six programs were excluded from the analysis; five were military programs, and one was not recruiting for the 2021-2022 cycle. Overall, 122 (87.8%) programs had Twitter accounts and 61 (43.9%) had Instagram accounts by the end of 2021, an increase from 88 (63.3%) and 12 (8.6%) in 2019, respectively (Table 1). Urology residency programs created 34 new Twitter accounts and 49 new Instagram accounts from 2019 to 2021. These new accounts constitute 28% of current urology Twitter accounts and 80% of current urology Instagram accounts. All Twitter and Instagram accounts were verified. There was no relationship between the AUA geographic section with the presence of Twitter (p=0.688) or Instagram (p=0.666) accounts.

Using the Shapiro-Wilk test, it was determined that the number of Twitter and Instagram posts was not normally distributed in 2019 for Twitter (p<0.001) but normally distributed for Instagram (p=0.456). Both Twitter and Instagram posts were not normally distributed in 2021 (p<0.001 and p<0.001, respectively). Of the programs that owned Twitter accounts in 2019 versus 2021, the median (interquartile range (IQR)) number of Tweets made by each program was 27 (44) versus 23 (51), respectively (Table 1). Programs posted on Instagram had a median (IQR) of 11.5 (22) times in 2019 and 13 (20) times in 2021 (Table 1). When comparing the 88 programs that owned Twitter accounts and the 12 programs that owned Instagram accounts in both 2019 and 2021, there was no significant change in the median number of posts between the two years for both Twitter (p=0.568) and Instagram (p=0.285) (Table 1).

**Table 1:** General Characteristics of Social Media (SoMe) Usage by Urology Residency Programs *Determined by account date creation for Twitter and earliest post for Instagram ^△^Significance determined using Mann-Whitney U test ^†^Statistical analysis only conducted between programs that owned respective accounts each year using Wilcoxon signed-rank test

	Twitter		Instagram	
Owned in 2019 (number (%))	88 (63.3%)		12 (8.6%)	
Owned in 2021 (number (%))	122 (87.8%)		61 (43.9%)	
Earliest account*	2009		2016	
Current # followers (median (interquartile range))	1079 (1198)		597 (397)	
Current # following (median (interquartile range))	307 (508)		106 (138)	
Doximity ranking: with SoMe, without SoMe (median)	67, 113	p<0.001^△^	66, 84	p=0.021^△^
Posts in 2019 (median (interquartile range))	27 (44)	p=0.568^†^	11.5 (22)	p=0.285^†^
Posts in 2021 (median (interquartile range))	23 (51)		13 (20)	

Association between Doximity ranking and social media 

**Figure 1:**
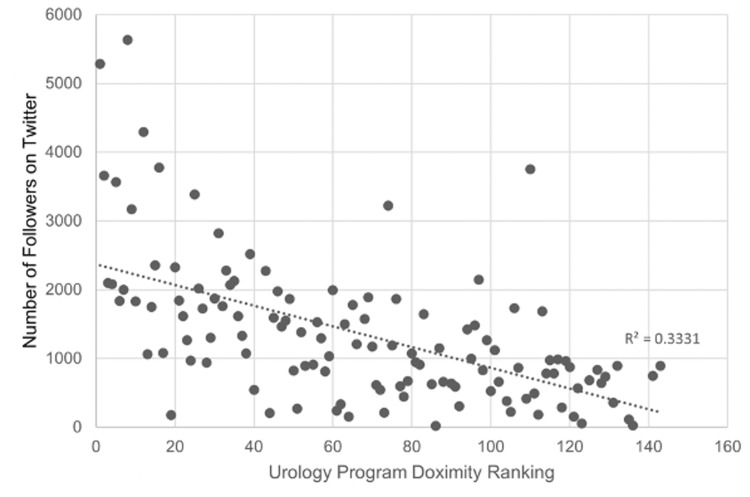
Number of Twitter Followers Versus Doximity Ranking

Urology residency programs with Twitter (n=122) had a median Doximity ranking of 67, whereas programs without Twitter (n=17) had a median Doximity ranking of 133 (Table 1). This difference was statistically significant (p<0.001). A significant linear regression was found based on a lower numerical Doximity rank (better ranking) and a higher number of Twitter followers (p<0.001) (Table 2, Figure 1). There was a significant linear regression between the Doximity ranking and the number of Twitter posts (p=0.005) (Table 2).

Urology residency programs with Instagram (n=61) had a median Doximity ranking of 66, whereas programs without Instagram (n=78) had a median Doximity ranking of 84 (Table 1). This difference was statistically significant (p=0.021). A significant linear regression was found based on Doximity ranking and the number of Instagram followers (p=0.026) (Table 2). There was no significant relationship between Doximity ranking and the number of Instagram posts (p=0.555).

**Table 2:** Linear Regression Results Between Social Media Metrics and Doximity Ranking *Statistically significant at p<0.05

		F (df regression, df residual)	F-value	R^2^-value	p-value
Twitter	Followers	F(1,120)	59.933	0.333	<0.001*
	Posts	F(1,120)	8.207	0.064	0.005*
Instagram	Followers	F(1,59)	5.204	0.081	0.026*
	Posts	-	-	-	0.555
Virtual Events		F(1,137)	7.717	0.054	<0.006*

Virtual events

In the application year 2021-2022, 107 (77%) urology programs hosted virtual residency recruitment events. For the programs that hosted virtual events, the median (IQR, range) number of hosted events was 2 (1, 1-6). A significant linear regression relationship was found between a program’s Doximity ranking and the number of virtual events (p<0.006) (Table 2).

## Discussion

In our study, we found that urology residency programs that had Twitter or Instagram accounts tended to have a higher Doximity ranking. There was a significant relationship between a program’s Doximity ranking and the number of Twitter posts, Instagram followers, and virtual recruitment events, but the strongest correlation was with the number of Twitter followers.

Since the start of the COVID-19 pandemic, residency interviews transitioned virtually, and sub-internships were greatly limited during both the 2020-2021 and 2021-2022 AUA Match cycles [18-20]. Many programs and applicants increased their social media presence and relied on these virtual avenues for residency recruitment [3,6]. This study investigated social media activity by urology residency programs between 2019 and 2021, virtual residency recruitment events during the 2021-2022 AUA Match cycle, and programs’ Doximity rankings. While this study affirms the gaining popularity of social media by urology programs as seen in prior studies [3,5,6], this is the first study demonstrating a relationship between a urology residency program’s social media activity and Doximity rankings.

There was a large increase in Twitter and Instagram account creation by urology residency programs from 2019 to 2021, which is consistent with previous literature [5]. This trend was seen in other specialties such as general surgery, with a threefold increase in Twitter account creation and a nearly sevenfold increase in Instagram account creation when comparing 2020 with 2019 [21]. Similarly, for the residency programs that had an Instagram account, 85% of orthopedic surgery accounts and 88% of pathology accounts were created in 2020 [10,22]. However, when comparing urology programs that had existing social media accounts since 2019 or earlier, our study did not find any significant difference in posting activity on Twitter or Instagram in 2019 versus 2021. Therefore, the COVID-19 pandemic may have influenced the number of new social media accounts created by urology programs but not the amount of social media activity. A program’s stronger social media presence may generate a more favorable perception through this increased public visibility and popularity. This may result in a program receiving more positive resident and attending faculty survey responses, which are utilized to partially determine a residency program’s Doximity ranking. Another possibility is that more prestigious programs may have the resources to fund nonclinical staff who could manage and contribute more actively on social media.

There was a significant relationship between a program’s Doximity ranking and the number of Twitter followers, Twitter posts, and Instagram. However, this relationship had the strongest correlation between a program’s Doximity ranking and Twitter followers. Our findings echo that of an AUA Match survey in 2021 that reported that 84% of applicants found Twitter to be helpful versus only 14% of applicants for Instagram [6]. Although Instagram has been found to be the fastest-growing social media among urologists [4], it is hypothesized that Instagram has not gained the same reputation as Twitter as a “reputable, professional, and academic” network [6]. To further support this hypothesis of Twitter as a professional social media platform, the use of Twitter by urology departments and urological journals has been associated with a more prestigious U.S. News and World Report (USNWR) ranking and higher impact factor, respectively [2,23,24]. These findings suggest that social media usage may continue to have a growing impact on the future of academic urology. If future AUA Match cycles continue to be virtual, urology programs should consider increasing their Twitter and Instagram presence to not only assist with residency recruitment but to also potentially increase Doximity ranking.

There was a significant relationship between a program’s Doximity ranking and the number of virtual recruitment events. An overwhelming majority (77%) of urology residency programs hosted virtual recruitment events during the 2021-2022 AUA Match cycle, compared to 75% of orthopedic surgery [25], 59% of anesthesiology [26], 30% of neurosurgery [27], and 25% of pathology programs [22] in 2020-2021. Applicants reported that these virtual events could be used to distinguish between programs, discuss strengths and weaknesses, and participate in resident question-and-answer sessions [7]. Not only do these virtual recruitment events provide valuable opportunities for applicants, but we believe they may also be an important tool for programs to gauge initial interest from prospective residents [7].

The main limitation of our study is the relative paucity of metrics to rank urology residency programs. Our study relied on the Doximity reputation rankings, which are based on three components: satisfaction data via current resident and alumni surveys, reputation data via board-certified physician surveys, and objective data via research output and a “proprietary Doximity database” [13]. While it is likely that these factors will reflect academic prestige, Doximity’s survey methodology has been criticized as too “subjective” and potentially biased by the size of the program, as a larger program may encourage more survey responses [8,28]. The USNWR ranking was considered a possible metric, although ultimately not utilized, as this ranking more accurately reflects the prestige of urology departments only, rather than in addition to residency programs [23]. Another limitation of our study is that we did not collect data for Facebook. Although popular, Facebook was omitted because previous literature has consistently shown it to be much less popular than Twitter and Instagram for urology residency recruitment [6,10].

A final comment is that our study does not imply that social media is the only factor in rankings. With urology’s highly competitive environment in both education and practice, many variables determine academic prestige, and social media may be one of many. The goal of this study is primarily to voice our recommendations by providing additional data for urology programs to make informed decisions on their own social media activity.

More research is needed to investigate the relationship between social media usage by urology residency programs and their academic prestige. A future direction of interest would be to analyze the content of Twitter and Instagram posts, particularly how many of these posts were used for residency recruitment specifically. Although Tweet categories (e.g., guidelines, awards, self-promotion, and research) have been examined in prior studies, it was not done so in relation to a program’s ranking [23]. Analyzing the relationship between Doximity rankings and retweets, replies, and comments could be another future area of interest as this current study only looked at primarily authored Tweets. Lastly, additional research exploring the validity and mechanism of Doximity rankings is also needed.

## Conclusions

The use of social media by both urology residency programs and applicants has been largely increasing as a result of the COVID-19 pandemic requiring residency recruitment and interviews to become primarily virtual. There was a significant relationship between a program’s Doximity ranking and the presence of Twitter or Instagram, as well as the number of Twitter followers, Twitter posts, Instagram followers, and virtual recruitment events. Although Twitter appears to be more widely utilized and a better indicator than Instagram regarding academic prestige on Doximity, both platforms have been used by the majority of urology residency programs.​
